# Emergency medicine residents and performance under pressure: learning from elite athletes’ experience

**DOI:** 10.1186/s12245-024-00648-8

**Published:** 2024-05-21

**Authors:** Gabrielle Trepanier, Viviane Falardeau, Gurpreet Sohi, Veronique Richard

**Affiliations:** 1 Department of Family Medicine and Emergency Medicine, Faculty of Medicine and Health Sciences, University of Sherbrooke, 3001, 12E Avenue Nord, Sherbrooke, Québec J1H 5N4 Canada; 2 Faculty of Medicine and Health Sciences, University of Sherbrooke, Sherbrooke, Québec Canada; 3 Faculty of Medicine and Health Sciences, University of British Columbia, Kelowna, BC Canada; 4 School of Human Movement and Nutrition Sciences, University of Queensland, Brisbane, Australia

## Abstract

**Objective:**

The skills of coping with stress and pressure within emergency medicine are conveyed informally and inconsistently throughout residency training. This study aims to identify key psychological competencies used by elite athletes in high-pressure situations, which can be integrated into a formal curriculum to support emergency medicine residents’ performance in high acuity settings.

**Design:**

We conducted a scoping review spanning 20 years to identify the relevant psychological competencies used by elite athletes (Olympic or World level) to perform under pressure. We used controlled vocabulary to search within Medline, PsycInfo and SportDiscuss databases. A standardized charting method was used by the team of four authors to extract relevant data.

**Results:**

The scoping review identified 18 relevant articles, including 707 athletes from 49 different sports and 11 countries, 64 data items were extracted, and 6 main themes were identified. The main psychological competencies included the ability to sustain a high degree of motivation and confidence, to successfully regulate thoughts, emotions and arousal levels, and to maintain resilience in the face of adversity.

**Conclusion:**

We used the main psychological competencies identified from our scoping review to develop a hypothesis generated framework to guide the integration of performance psychology principles into future emergency medicine residency programs.

**Supplementary Information:**

The online version contains supplementary material available at 10.1186/s12245-024-00648-8.

## Introduction

The emergency department can be high-pressure, crowded environment where the consequences of impaired physician performance can be severe. Although pressure can impair performance [1–3], the psychological competencies required to manage stress in high-pressure situations are not taught in a formal curriculum in emergency medicine residency programs. Consequently, a subset of emergency physicians develop maladaptive coping strategies that can lead to mental health challenges, addictions and physician burnout [4–6]. Research indicates that reducing psychological distress in emergency physicians leads to improved mental health outcomes, enhanced well-being, prolongs careers [7, 8] and promotes patient safety [9]. The current demand for this curriculum is particularly high as emergency physicians have faced unprecedented psychological adversity over the COVID-19 pandemic [10], staff shortages [11], and resource limitations [12].

Various fields such as the military, aviation, and music industries have explored the use of psychological skills for performance under stress, yet the increasing relevance of sports psychology, particularly in elite sports, captures our interest. Olympic athletes, for instance, cultivate psychological competencies that allow them to consistently excel under extreme pressure [13]. Our focus is to identify the psychological competencies employed by elite athletes in high-stress environments that could be beneficial in developing a pedagogical curriculum.

Despite a growing interest in applying the principles of sport psychology to health profession education [14–16], there has been limited research into its applicability to training emergency physicians. To bridge the gap, this scoping review aims to identify psychological competencies associated with performing under pressure in elite athletes, and to explore how these can be transferred to optimize performance in emergency medicine residency training.

## Methodology

A scoping review of the last 20 years of literature was conducted in April 2023, according to the PRISMA-ScR methodology [17] to explore the psychological competencies employed by elite athletes performing under pressure. This methodology was chosen to identify the available evidence in a specific field and key characteristics associated with a concept, as described by Munn [18] and supported by Grant and Booth's typology of reviews [19]. The research team consisted of four investigators: an emergency physician and emergency program director (GT), an emergency physician responsible for the well-being curriculum in the same program (VF), a Doctor of Sport Sciences—Performance Psychology (PhD) (VR) and an Olympic athlete and current medical student (GS).

The key words and controlled vocabulary (Medical Subject Headings or MESH terms) relevant to the subject were chosen by the two principal authors (GT and VR) with the help of a specialized librarian. Articles were identified in the following databases: MEDLINE, PsycINFO and SPROTDiscuss. Search strategies and keywords are available in the additional materials section (Appendix I). Studies that were not relevant or not in English were excluded.

All articles retrieved from the databases were imported into EndNote, and duplicates were eliminated. Titles and abstract were screened for the following eligibility criteria:

1. Athletes and para-athletes had to perform at the Olympic level, International level and/or World level;
2. Psychological competencies had to be linked to performance under pressure;
3. Original studies published by a peer review journal

**Fig. 1:**
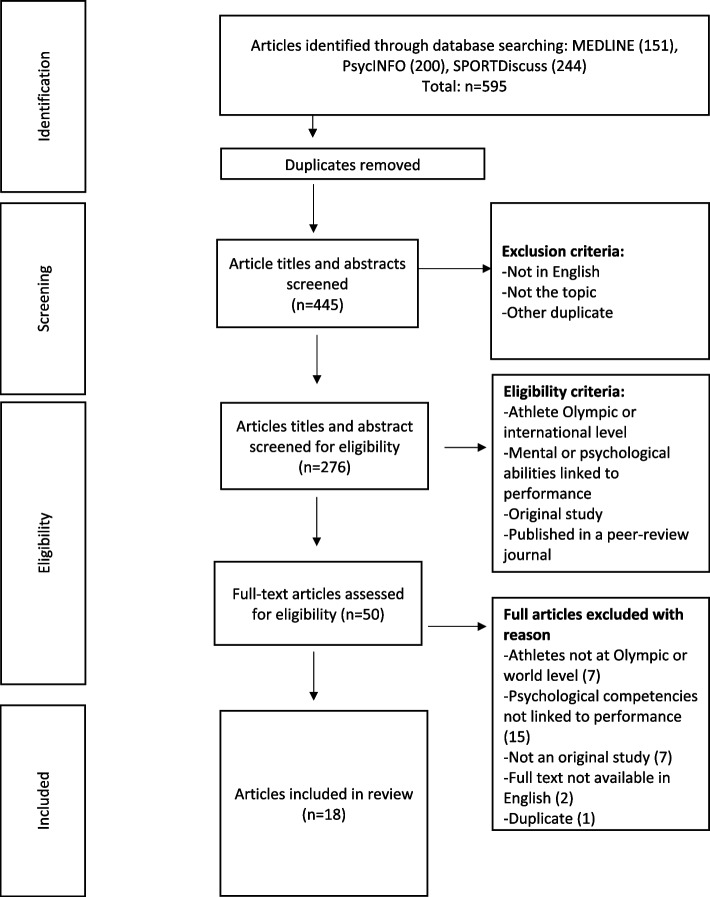
PRISMA flow diagram describing research strategy

The inclusion of each article was discussed among the four authors after full-text assessment. Fifteen articles were accepted for the review based on initial consensus. Eleven articles were discussed among authors and eventual consensus was reached to include three additional articles based on the inclusion criteria. A total of 18 articles were included in the scoping review (see Fig. 1 PRISMA) and proceeded through the data-charting process.

A customized Excel form developed by the primary authors (GT and VR) was utilized to systematically collect data from the chosen articles. To ensure accuracy and consistency, all four authors engaged in a calibration exercise of five sample articles. The screening was done in duplicate (i.e., two reviewers independently) and then reviewed at a team meeting.

The extracted results were subsequently grouped by similar psychological competencies and demographic information was collected from the articles. Data items were independently extracted by two members and later shared with the rest of the author team for cross-validation, confrontation, and further discussion in an iterative process. The final set of citations was determined, focusing on the identified themes related to psychological competencies in an iterative process.

## Results

A total of 18 papers were retrieved: seven qualitative studies, ten quantitative, and one of mixed methods. A total of 707 elite athletes were included, with a mean age of 24 years, representing 49 different sports and 11 countries. Table 1 presents the main details of the studies and Table 2 the details of each article.

**Table 1:** List of the principal psychological competencies for each article

Article’s title	Authors	Number of data items	Themes
Investigating the Optimal Psychological State for Peak Performance in Australian Elite Athletes	Anderson, Ruth Hanrahan, Stephanie J Mallett, Clifford J	8	Confidence / self efficacy Regulation of thoughts Regulation of emotions Resilience
Mental and physical attributes defining world-class Norwegian athletes: Content analysis of interviews	Boes, R., Harung, H.S., Travis, F. & Pensgaard, A.F	3	Motivation / commitment Regulation Resilience
Lifestyles and mindsets of Olympic, Paralympic and world champions: is an integrated approach the key to elite performance?	Burns, L. Weissensteiner, J.R. & Cohen, M	9	Motivation / commitment Confidence / self efficacy Regulation of thoughts Resilience
Dealing with elite sport competition demands: An exploration of the dynamic relationships between stress appraisal, coping, emotion, and performance during fencing matches (study 1)	Doron, J. & Martinent, G	2	Regulation of thoughts Regulation of emotions
Dealing with elite sport competition demands: An exploration of the dynamic relationships between stress appraisal, coping, emotion, and performance during fencing matches (study 2)	Doron, J. & Martinent, G	2	Regulation of thoughts Regulation of emotions
Expected and Unexpected Stressors in Major International Competition: Appraisal, Coping, and Performance	Dugdale, J.R., Eklund, R.C. & Gordon, S	1	Regulation of thoughts
A grounded theory of psychological resilience in Olympic champions	Fletcher, D & Sarkar, M	5	Motivation / commitment Confidence / self efficacy Regulation of thoughts Resilience
Higher psycho-physiological refinement in world-class Norwegian athletes: brain measures of performance capacity	Harung, H.S., Travis, F., Pensgaard, A.F., Boes, R., Cook-Greuter, S. & Daley, K	5	Regulation of thoughts Regulation of emotions
The role of confidence in world class sport performance	Hays, K., Thomas, O., Maynard, I. & Bawden, M	3	Motivation / commitment Confidence / self efficacy Regulation of thoughts Regulation of emotions
What makes an orienteer an expert? A case study of a highly elite orienteer’s concerns in the course of competition	Macquet, A-C., Eclles, D.W. & Barraux, E	5	Regulation of thoughts
Anxiety Characteristics of Competitive Windsurfers: Relationships with Age, Gender, and Performance Outcomes	Modroño, C. & Guillen, F	2	Confidence / self efficacy Regulation of emotions Regulation of arousal
Sydney 2000: The Interplay Between Emotions, Coping, and the Performance of Olympic-Level Athletes	Pensgaard, A.M. &Duda, J.L	2	Regulation of emotions Regulation of arousal
Influence of cognitive interferences and self-talk functions on performance during competition in elite female field hockey players	Pérez-Encinas, C., Fernández-Campos, F.J. & Barrios, J.R.C	2	Regulation of thoughts
Mental skill profiles and expertise levels of elite Iranian athletes	Monfared, S. S., Mosayebi, F. & Durand-Bush, N	2	Regulation of thoughts Regulation of emotions
Pre-performance psychological states and performance in an elite climbing competition	Sanchez, X, Boschker, M.S.J. & Llewellyn, D.J	3	Confidence / self efficacy Regulation of thoughts Regulation of emotions Regulation of arousal
Self-efficacy, flow, affect, worry and performance in elite World Cup ski jumping	Sklett, V.H., Lorås, H.W. & Sigmundsson, H	4	Confidence / self efficacy Regulation of emotions Regulation of arousal
Self-regulation of elite athletes in china	Sun, Y. & Wu, X	3	Regulation of thoughts Regulation of emotions
Self-confidence, commitment and goal-setting in Czech athletes at different performance levels	Vičar, M	3	Motivation / commitment Confidence / self efficacy

**Table 2:** Characteristics of each article regarding authors, country, year of publication, number of athletes, methodology of the article, strenghths, and limitations of the study

Title	Authors	Country	Year of publication	Number of athletes	Methodology	Strengths	Limitations
Investigating the optimal psychological state for peak performance in Australian elite athletes	Anderson, R., Hanrahan, S.J. & Mallet, C.J	Australia	2014	17	Semi structured Interview, qualitative	Directly linked "peak performance" with concept of automaticity	Biases due to self-reporting
Mental and physical attributes defining world-class Norwegian athletes: Content analysis of interviews	Boes, R., Harung,H.S., Travis, F. & Pensgaard, A.F	Norway	2014	28	Semi structured Interview, qualitative	Mixed methodology Used a control group Broad holistic approach	All inner/outer variables found non-significant
Mental and physical attributes defining world-class Norwegian athletes: Content analysis of interviews	Burns, L. Weissensteiner, J.R. & Cohen, M	Australia	2014	10	Semi structured Interview, qualitative	Included paralympic athletes Sample includes only very high performing athlete	Small sample size from different sports Generational differences between athletes
Dealing with elite sport competition demands: An exploration of the dynamic relationships between stress appraisal, coping, emotion, and performance during fencing matches (**studies 1 and 2**)	Doron, J. & Martinent, G	France	2021	16	Multi-sectioned questionnaire, quantitative	Ecological experiment	Simulation Small sample size Single item approach
Expected and unexpected stressors in major international competition: appraisal, coping, and performance	Dugdale, J.R., Eklund, R.C. & Gordon, S	Australia	2002	91	Multi-sectioned questionnaire and open-ended questions, mixed methods	Questionnaires administered immediately after competition Questioned a specific event (not a hypothetical) Use of self-reference performance indices	Retrospective study Lack of scientific rigor
A grounded theory of psychological resilience in Olympic champions	Fletcher, D & Sarkar, M	UK	2012	12	Semi structured interview, qualitative	Supra elite athletes Theoretical model grounded in original data	Retrospective Uncertain validity of the linear stage model
Higher psycho-physiological refinement in world-class Norwegian athletes: brain measures of performance capacity	Harung, H.S., Travis, F., Pensgaard, A.F., Boes, R., Cook-Greuter, S. & Daley, K	Norway	2011	33	EEG, quantitative; Experimental—cross sectional	Rigorously selected control group EEG is an objective measure	EEG during non-specific task – less transferable
The role of confidence in world class sport performance	Hays, K., Thomas, O., Maynard, I. & Bawden, M	UK	2009	14	Semi structured interview, qualitative	Strong sample of athletes, level athlete In depth exploration role of confidence	Retrospective study Gender differences in reporting symptoms of anxiety and self- confidence might account for the results
What makes an orienteer an expert? A case study of a highly elite orienteer's concerns in the course of competition	Macquet, A-C., Eclles, D.W. & Barraux, E	France & USA	2012	1	Semi structured interview, qualitative (case–control)	Rare study of cognitive activity of expert orienteerer Rare study of an athlete's concerns in relation to entire competitive events Highly elite status athlete with prototypical value Video analysis and interview	Case study No non expert controls Participant asked to comment on activity seen in video film, so excludes input of other senses, inherent to orienteering
Anxiety characteristics of competitive windsurfers: relationships with age, gender, and performance outcomes	Modroño, C. & Guillen, F	Spain	2011	54	Multi-sectioned questionnaire, quantitative	Research conducted by sports psychologists	All female sample
The interplay between emotions, coping, and the performance of Olympic-level athletes	Pensgaard, A.M. &Duda, J.L	UK	2000	61	Multi-sectioned questionnaire, quantitative	Results similar to previous research	Retrospective Limited response rate Recall bias
Influence of cognitive interferences and self-talk functions on performance during competition in elite female field hockey players	Pérez-Encinas, C., Fernández-Campos, F.J. & Barrios, J.R.C	Spain	2016	20	Multi-sectioned questionnaire, quantitative	n/a	Small sample size Retrospective Raking may taint responses
Mental skill profiles and expertise levels of elite Iranian athletes	Salmela, J. H., Monfared, S. S., Mosayebi, F. & Durand-Bush, N. (	Islamic Republic of Iran & Canada	2009	214	Multi-sectioned questionnaire, quantitative	n/a	Specifc to Iranian population
Pre-performance psychological states and performance in an elite climbing competition	Sanchez, X, Boschker, M.S.J. & Llewellyn, D.J	Denmark	2010	19	Multi-sectioned questionnaire, quantitative	First ecologically valid study of pre-performance psychological states and performance in elite climbers	Evaluation only of emotional states before the competition Small all-male sample
Self-efficacy, flow, affect, worry and performance in elite World Cup ski jumping	Sklett, V.H., Lorås, H.W. & Sigmundsson, H	Norway	2018	40	Multi-sectioned questionnaire, quantitative	n/a	Single competition evaluation No quantitative data collected Scales did not account for important variables like snow conditions and prior injury
Self-regulation of elite athletes in China	Sun, Y. & Wu, X	China	2011	14	Semi structured interview, qualitative	n/a	Retrospective Many factors implicated in performance, in relation to suboptimal performance, were not evaluated
Self-confidence, commitment and goal-setting in Czech athletes at different performance levels	Vičar, M	Czech Republic	2018	63	Multi-sectioned questionnaire, quantitative	n/a	Lack of power Probable cultural differences in reporting Only used 3/12 of the OMSAT-3 scales

The team extracted a total of 64 data items. Six main themes emerged on psychological competencies: 1) motivation and commitment, 2) confidence and self-efficacy, 3) regulation of thought and attention, regulation of emotion, regulation of arousal and 4) resilience (see Table 2). The principal findings are presented in a narrative format.

### Psychological competencies enhancing performance under pressure

#### Motivation and commitment

High levels of motivation and commitment are key to supporting elite athletes’ performance under pressure [20–24]. At the highest level of performance, athletes’ motivation and commitment can take the form of a self-reliant desire to improve and grow [20], a strong passion for the sporting task [21], an insatiable hunger for challenges [21, 24] and a drive to perform successfully or to be the best [22]. The capacity to deliberately choose to engage with challenging situations is particularly relevant when it comes to facing adverse situations. It allows athletes to positively appraise challenges, increasing the likelihood of athletes approaching these challenges rather than avoiding them [24].

#### Confidence and self-efficacy

For athletes to achieve peak performance when the stakes are high, confidence is an invaluable asset [21–25]. Athletes’ belief in their ability to successfully achieve goals positively impacts their thoughts, feelings, and behaviors [21, 22], leading to more effective assessment of stressors [24]. Specifically, high confidence levels can help athletes focus effectively, interpret their “nerves” as excitement rather than fear [22], and perform optimally in competition [26–28]. Multiple sources of confidence have been identified to facilitate the performance of elite athletes such as preparation, experience, self-awareness, visualization, coaching and social support [24]. Contrarily, factors such as poor performance, injury/illness, pressure and great expectations can harm confidence leading to a future drop in performance [22].

#### Regulation and adaptability

Frequently referred to as *coping*, regulation of thoughts, emotions, and arousal to manage exceedingly demanding situations, is central to positive performance outcomes [25, 28–30]. Through self-reflection and self-awareness of their strengths and limitations [21], elite athletes develop regulation competencies to achieve the right combination of conditions to support automatic skill execution [25]. Stress appraisal and perception of control over a situation can impact the type of coping used by athletes [31]. Moreover, stress control was found to significantly differentiate between medallists and non-medallists in an international competition [32].

##### Regulation of thought and attention

At the cognitive level, the ability to appraise situations as a challenge rather than a threat [24, 31] and regulate attention to focus on the task at hand [20, 22, 30] enhances performance under pressure. Specifically, *challenge appraisal* occurs when athletes evaluate their resources as sufficient to tackle the demand of a stressful situation [24]. It is important to note that the type of stressor impacts appraisal. Namely, athletes tend to appraise unexpected stressors as more threatening than expected ones, leading to hesitation to act [33]. This can be explained by athletes’ need to perceive control over a stressful situation in order to deploy appropriate coping strategies to perform optimally [31]. The appraisal of a situation as too challenging and/or the lack of perceived control may generate doubts in an athlete’s mind leading to performance deterioration [28].

Interestingly, higher EEG coherence in frontal executive systems of elite athletes compared to less successful ones indicate that their capacity to excel in sport is supported by a superior capacity to perceive, plan, and strategize [34]. In addition, the ability to refocus attention was found to be higher among athletes selected for international-level competitions [32]. Furthermore, performance under pressure improves when athletes exhibit the capacity to flexibly switch between strategies and make effective decisions under complexity, uncertainty, and time pressure [35]. Finally, the ability to control interfering thoughts was found to reduce internal disturbance leading to higher quality sporting outcomes [36].

##### Regulation of emotion

High pressure situations can trigger a myriad of emotions; thus, emotional regulation is crucial to achieve an optimal performance state [25, 28, 30]. Contrary to the assumption that predominantly positive emotions contribute to this state (e.g., being calm), findings reveal that experiencing both positive and negative emotions in the lead up to performance is beneficial [27, 29]. For instance, emotions such as anger, enthusiasm, anxiety, and relaxation were reported by elite athletes to be *optimizing emotions* while being scared, happy, determined, and tired were reported as *dysfunctional emotions.* Hence, to move beyond the positive vs negative dichotomy, it is suggested to refer to the emotion-performance relationship: negative and positive emotions must be further labeled as optimizing or dysfunctional, considering that not all positive emotions are optimizing and vice versa [29].

While some argue that problem-focused coping is preferable in managing dysfunctional emotions [31], others have shown that it is the perceived effectiveness of coping that significantly impacts the achievement of optimal emotional responses to boost performance in stressful situations [29]. In other words, the type of coping strategies used by athletes (e.g., problem-focused vs emotion-focused) is not relevant as long as they perceive that the effort invested to cope is effective enough to deal with the acute stressors [29]. Other psychological competencies such as confidence may also influence athletes’ capacity to cope with emotions [22, 28].

##### Regulation of arousal

The capacity to regulate arousal, often referred to as somatic anxiety, is the physiological component associated with a stress response. Considerably linked to cognition and emotion, regulation of arousal also impacts how individuals overcome challenging situations. Yet, the arousal-performance relationship remains unclear. While some findings revealed that lower levels of pre-competitive somatic anxiety are associated with better performance rankings [26], others suggest that higher levels positively correlate with performance outputs [27]. Thus, it may be that the arousal-performance relationship is idiosyncratic, and athletes must find their own balance to perform successfully. In line with this argument, EEG results revealed that elite athletes remained at a balance point between simultaneously being calm and alert during reaction time tasks [34].

#### Resilience

Specific psychological factors, cited previously, act as protective measures for elite athletes against the adverse effects of stress while also fostering adaptive responses in stressful situations [24]. When facing adversity, resilience promotes adaptability, the activation of coping mechanisms and the management of expectations [25]. Social support also plays a vital role in buffering stress and fostering resilience [21].

One distinguishing factor among elite athletes was their unwavering commitment to continuous improvement and personal growth. They constantly engaged in reflective thinking, focused on enhancing their skills, progress, and overall performance optimization [20, 21]. Although elite athletes may initially perceive a stressor negatively, they possess the ability to subsequently recognize the potential of the resulting emotion to enhance their performance [24]. Thus, highly resilient athletes possess the ability to restore an optimal psychological state during performance [25].

## Discussion

This study provides valuable insight from high-performance athletes worldwide, providing the potential of applying the identified psychological competencies to a broad range of individuals performing under stress, including emergency medicine physicians. Our findings are a step towards a more concise and practical approach to support training for emergency medicine residents.

### Pedagogic / comprehensive curriculum

**Fig. 2:**
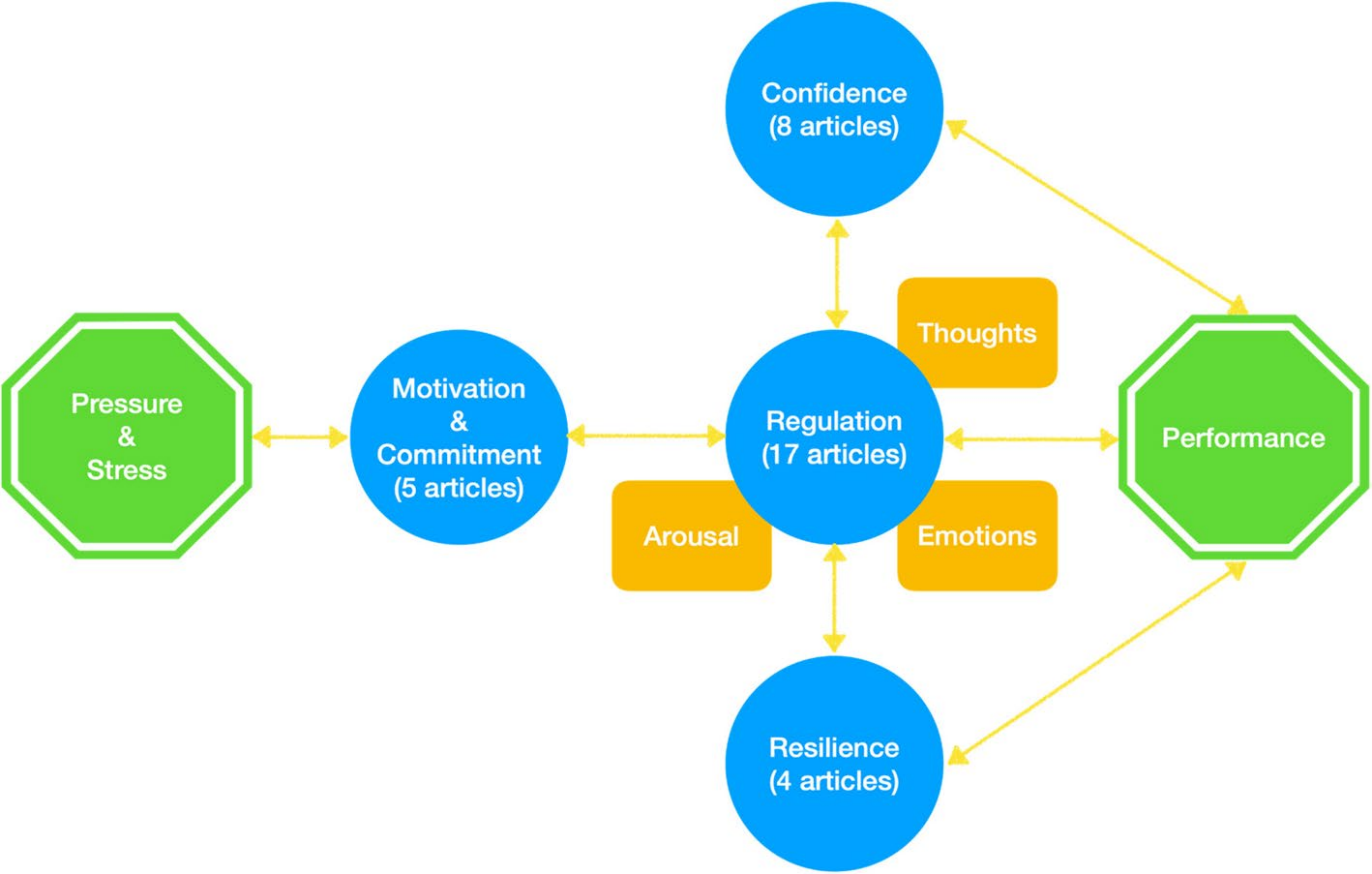
Hypothesis-generated framework to psychological competencies proposed by the authors, with the number of articles cited fo each one in brackets

While psychological competences are presented separately in the Result section, they are all interconnected in order to facilitate performance under pressure (see Fig. 2). As physicians face high-pressure situations, we assert that their levels of motivation and confidence vary, influencing their thoughts, emotions, and arousal level [20–24]. To increase the likelihood of successful performance, physicians must activate regulation competencies to reach their optimal performance states and adapt to circumstances [21, 25, 28–31]. When faced with additional challenges, resilience becomes crucial to maintain motivation and confidence [21, 24, 25]. Successfully overcoming challenges may, in turn, positively impact motivation and confidence, creating a positive psychological feedback loop [20, 21, 24, 25].

Because these competencies can be taught [37–40], this model provides a comprehensive framework for residency programs to integrate psychological training as a part of the curriculum. For instance, residents should receive training in areas such as motivation and commitment, emotional regulation, resilience, and confidence building. It also enables teachers to debrief challenging situations by addressing all six key psychological competencies with their residents.

### Previous studies

While several performance psychology models exist in the sports literature [13, 41–43], few empirical studies have explored how to develop mental competencies to support performance in general medicine training [14, 16, 37] or surgery training [15, 38–40, 44]. No studies were retrieved that directly address the development and transfer of these competencies to emergency medicine training. This study presents a concise and practical approach that can help emergency medicine residency programs build a curriculum to support the development of these psychological competencies.

### Strengths and limitations

This review identified studies that revealed psychological competencies that could be helpful in designing a psychological curriculum in an emergency medicine residency training program. The authors of the review come from diverse experiential backgrounds (pedagogical research, sports psychology, Olympic-level athletics) which facilitated extraction of a broader and more comprehensive review. Finally, this review was composed of studies including a significant number of elite athletes, with a mean age similar to that of emergency medicine residents in training.

However, studies included in the review were unequal in their methodological rigor which limits the applicability and half of them were qualitative studies exploring the psychological aspects of performing under pressure. Furthermore, although we appreciate the diversity in countries of origin for our article list, local application of recommendations from such a wide range of nations and cultures and may have practical challenges and limitations. Furthermore, only one study was conducted in North America, which could limit the applicability of their findings to a Canadian population. Consequently, our research is primarily exploratory and aimed at generating hypotheses, and the results should be interpreted within the context of these limitations.

### Clinical and research implication

The proposed model could help build a curriculum to develop psychological competencies among emergency medicine residents. This curriculum has the potential to better equip emergency physicians for clinical practice, increase staff retention and improve career longevity. The question remains: how do we best build these competencies in emergency medicine residents? Additional investigations are required to identify the best strategy to develop each of these competencies.

## Conclusion

Coping with stress and pressure is an essential mental skill of emergency physician to perform and optimize patient care in an emergency department setting. However, these skills are in often taught informally and inconsistently in residency programs. Mastering those psychological competencies is key to ensuring resiliency and career longevity. Therefore, the integration of psychological competencies identified from this scoping review into future emergency medicine residency programs could better prepare residents to mentally address day-to-day challenges in the emergency department, mitigate burnout and improve quality of care in high stressful circumstances.

### Supplementary Information

Supplementary Material 1.

## Data Availability

No datasets were generated or analysed during the current study.
