# Supplementary material for: Emergency medicine residents and performance under pressure: learning from elite athletes’ experience
Source: Int J Emerg Med. 2024 May 21;17:67. doi: 10.1186/s12245-024-00648-8 (PMC11106854; doi:10.1186/s12245-024-00648-8)

Research strategies

| Box 1 | Box 2 | Box 3 |
| --- | --- | --- |
| ( athlet* OR competition OR sport*) N2 (Olympi* OR world* OR international*) | ( stress OR pressure OR performance ) ) OR AB ( ( stress OR pressure OR performance ) | (psychologic* OR mental* OR coping OR cognitive) ) OR AB ( (psychologic* OR mental* OR coping OR cognitive) |

Sport Discuss


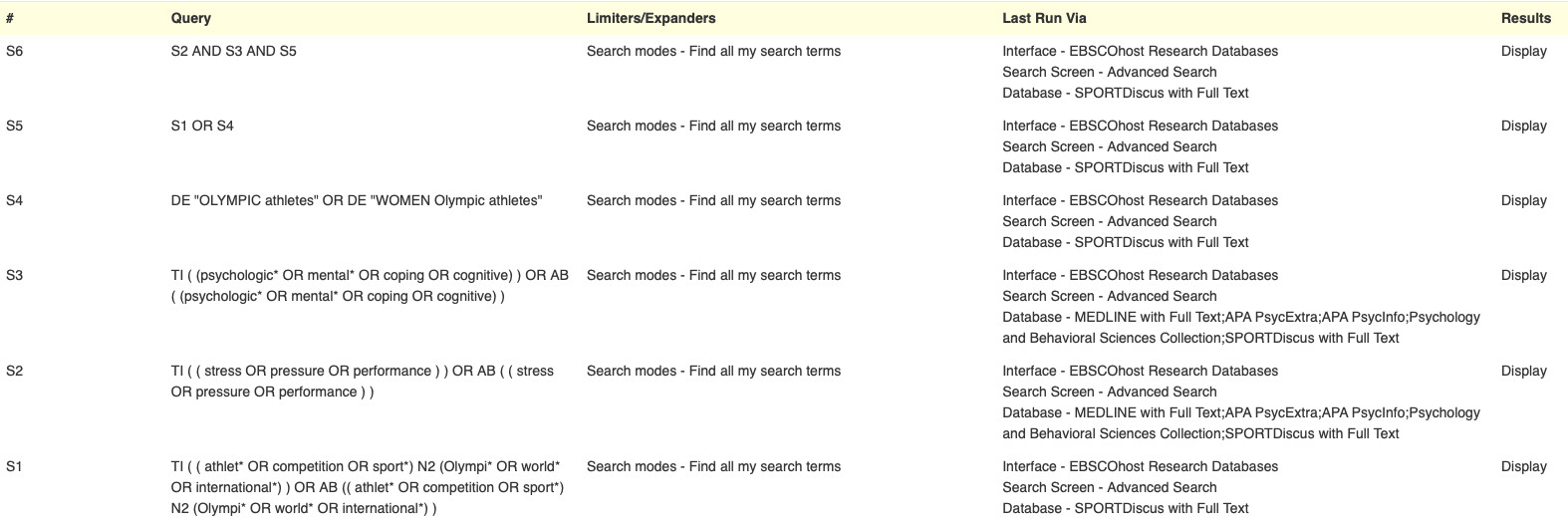


MEDLINE, APA PsycInfo


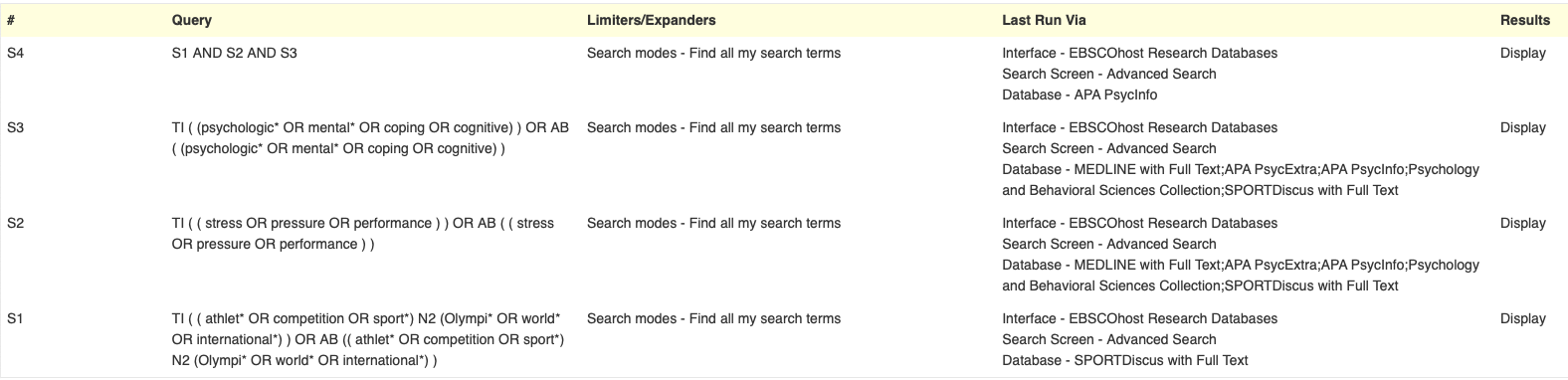

Supplement: Supplementary file 1 — Supplementary Material 1. [file 12245_2024_648_MOESM1_ESM.docx]
